# Self-Propagative Replication of Aβ Oligomers Suggests Potential Transmissibility in Alzheimer Disease

**DOI:** 10.1371/journal.pone.0111492

**Published:** 2014-11-03

**Authors:** Amit Kumar, Kayla M. Pate, Melissa A. Moss, Dexter N. Dean, Vijayaraghavan Rangachari

**Affiliations:** 1 Department of Chemistry and Biochemistry, University of Southern Mississippi, Hattiesburg, United States of America; 2 Department of Chemical Engineering, University of South Carolina, Columbia, United States of America; University of Essex, United Kingdom

## Abstract

The aggregation of amyloid-β (Aβ) peptide and its deposition in parts of the brain form the central processes in the etiology of Alzheimer disease (AD). The low-molecular weight oligomers of Aβ aggregates (2 to 30 mers) are known to be the primary neurotoxic agents whose mechanisms of cellular toxicity and synaptic dysfunction have received substantial attention in the recent years. However, how these toxic agents proliferate and induce widespread amyloid deposition throughout the brain, and what mechanism is involved in the amplification and propagation of toxic oligomer species, are far from clear. Emerging evidence based on transgenic mice models indicates a transmissible nature of Aβ aggregates and implicates a prion-like mechanism of oligomer propagation, which manifests as the dissemination and proliferation of Aβ toxicity. Despite accumulating evidence in support of a transmissible nature of Aβ aggregates, a clear, molecular-level understanding of this intriguing mechanism is lacking. Recently, we reported the characterization of unique replicating oligomers of Aβ42 (12–24 mers) *in* *vitro* called Large Fatty Acid-derived Oligomers (LFAOs) (Kumar et al., 2012, *J. Biol. Chem*). In the current report, we establish that LFAOs possess physiological activity by activating NF-κB in human neuroblastoma cells, and determine the experimental parameters that control the efficiency of LFAO replication by self-propagation. These findings constitute the first detailed report on monomer – oligomer lateral propagation reactions that may constitute potential mechanism governing transmissibility among Aβ oligomers. These data support the previous reports on transmissible mechanisms observed in transgenic animal models.

## Introduction

Accumulating evidence indicates that pre-fibrillar aggregates, specifically the low-molecular weight oligomers of Aβ peptide, are responsible for the synaptic dysfunction and neuronal loss that occur in Alzheimer disease (AD) pathology [1]–[4]. Besides cellular toxicity, the widespread deposition of Aβ aggregates that occurs prior to neuronal death, as observed in both post-mortem AD brains and transgenic (Tg) mice brains overexpressing Aβ, has generated significant curiosity during the last few years. Recent data indicate that the inoculation of Aβ aggregate-laden brain homogenates into aggregate-free Tg AD mice brains overexpressing wild-type Aβ results in substantially increased Aβ deposition, suggesting an involvement of a seed-induced deposition mechanism [5]–[7]. More importantly, such inoculations not only induce deposition of aggregates at the site of injection but also at distant locations [5], [7]–[10], implicating the possibility that the dissemination of aggregates could occur by a mechanism similar to mammalian prion-type propagation. Such a mechanism involves a ‘template-assisted corruptive’ propagation of pathological conformers to quantitatively high amounts leading to dissemination of toxic species. It has also come to light that in addition to endogenous homogenates containing Aβ seeds, certain synthetic Aβ aggregates, when injected into Tg mouse brains, could induce widespread amyloid deposition similar to prions [11]. In a more recent study, distinct strains of Aβ prions demonstrated high fidelity of propagation upon serial passage in mice [12]. Reports such as these demonstrated for the first time that both endogenous and exogenous Aβ aggregates can be transmissible *in* *vivo* and support the growing thought that Aβ aggregates may act like disease-causing prions.

Along these lines, several recent pieces of evidence suggest that a common, prion-type mechanism may underlie many neurodegenerative diseases, confirming a long-held speculation based on their pathogenic similarities [5], [13]–[16]. The process of self-propagation is well known among mammalian prion diseases, of which the most common include Creutzfeldt-Jakob disease (CJD) in humans and Bovine spongiform encephalopathy (BSE) in livestock. In these diseases, the non-toxic cellular prion protein, PrP^C^, undergoes conformational changes to a misfolded, infectious scrapie form, PrP^Sc^. PrP^Sc^ in turn acts as a seed to convert more PrP^c^ to a similar infectious form leading to aggregates of PrP^Sc^ in a template-assisted manner [17]. This ‘protein only’ hypothesis of prion infectivity was first introduced by Griffith in 1967 [18] and has been consolidated by numerous recent reports. It is now believed that a similar protein corruptive mechanism may be also involved in the pathophysiology of other neurodegenerative disorders like Parkinson’s disease (PD), frontotemporal lobar degeneration (FTLD), and amyotophic lateral sclerosis (ALS), in addition to AD. Desplats and coworkers have shown that α-synuclein (αS), which is involved in PD, can migrate, infect neighboring neurons, and form Lewy bodies, suggesting a prion-like propagation mechanism [19]. A more recent report shows that extracellular αS can enter cells by endocytosis and act as a seed to promote the aggregation of intracellular αS in mouse model, further indicating the involvement of prion-like corruptive propagation [20]. Similar behavior has also been reported for superoxide dismutase (SOD1) and Tar DNA binding protein (TDP43) involved in ALS and FTLD, respectively [21]–[23]. In AD, replication of oligomers by self-propagation is relatively new and underexplored. Typically, replication would involve quantitative amplification of oligomers via monomer – oligomer or oligomer – oligomer interactions that may occur at the cost of fibril formation. So far, only a few *in* *vitro* oligomers such as fibrillar oligomers (FOs) and prefibrillar oligomers (PFOs) have been reported to undergo replication [24], [25].

Despite an increasing number of reports that support the possibility of Aβ replication by self-propagation *in* *vivo*, a paucity exists regarding the mechanistic understanding of such a mechanism *in* *vitro*. Furthermore, it is uncertain whether the property of replication is specific only to a few conformational strains of Aβ oligomers. So far, no oligomeric species has been identified as a bona fide physiological oligomer whose physiochemical properties are well characterized. One of the main reasons for this paucity of knowledge is the difficulty in generating and characterizing Aβ oligomers both *in* *vivo* and *in* *vitro*. Recently, we reported the generation and isolation of discrete a 12–24 mer oligomeric species of Aβ42 *in* *vitro*, called Large Fatty Acid-derived Oligomers (LFAOs) [26], [27]. We also demonstrated that LFAOs replicate upon interacting with Aβ42 monomers by a self-propagative mechanism [27]. However, several questions remained unanswered regarding the physiochemical and cellular properties of these Aβ oligomers. In this report, we have examined the behavior of LFAOs in cell culture and have investigated the molecular mechanism of LFAO replication. Our data suggests that LFAOs show physiological activity and undergo self-propagative replication by interacting with monomers. The effects of experimental conditions such as seed concentration and temperature on this process are presented and discussed. Together, the data demonstrate that LFAOs undergo replication that is dependent on seed concentration and temperature.

## Materials and Methods

### Materials

Wild-type Aβ42 was synthesized by the Peptide Synthesis Facility at the Mayo Clinic (Rochester, MN) using routine Fmoc chemistry. MALDI-TOF mass spectrometry revealed >90% purity of both peptides. SDS and thioflavin-T (ThT) were procured from Sigma (St. Louis, MO). Lauric acid (C12∶0) was purchased as sodium salt from NuCheck Prep, Inc. (Elysian, MN). Monoclonal Ab9 or Ab5 antibody specific for Aβ1-16 was supplied by Mayo Clinic Jacksonville, FL. The conformation specific polyclonal OC antibody was procured from Millipore, Inc. The Superdex-75 HR 10/30 size exclusion chromatography (SEC) column was purchased from GE Life Sciences. The gel electrophoresis and blotting instruments and buffers were procured from Bio-Rad Laboratories, Inc. (Hercules, CA). All other chemicals were obtained from VWR, Inc.

### Preparation of Aβ42 monomers

Lyophilized stocks of synthetic Aβ42 were stored at −20°C, desiccated. Briefly, 1.5–2.0 mg of peptide was dissolved in 0.5 mL of 35 mM NaOH and was allowed to stand at 25°C for 15 min prior to fractionation on a Superdex-75 HR 10/30 SEC column (GE Life Sciences) attached to an AKTA FPLC system (GE Healthcare, Buckinghamshire) to remove any preformed aggregates as previously reported [26]. The SEC column was pre-equilibrated in 20 mM Tris-HCl (pH 8.0) at 25°C and was run at a flow rate of 0.5 mL/min. One-minute fractions were collected. Concentrations of Aβ fractions were determined by UV-Vis spectrometry on a Cary 50 spectrophotometer (Varian, Inc.) using a molar extinction coefficient of 1450 cm^−1^ M^−1^ at 276 nm (www.expasy.org), corresponding to the single tyrosine residue in Aβ42. Periodically, the peptide purity after SEC was confirmed by MALDI-TOF mass spectrometry, which showed a monoisotopic molecular mass of 4516.31 Da. Monomeric Aβ42 fractions were stored at 4°C and used within 1–3 days.

### Aβ Aggregation Reactions

#### Propagation of LFAOs and globulomers

LFAOs and globulomers were generated based on previously reported protocols [27], [28]. Monomeric Aβ42 (20 µM) was incubated for 72 h in 20 mM Tris pH 8.0 at 25°C alone or with 2% (molar ratio) LFAOs or 2 and 5% (molar ratio) Aβ globulomers seeds. Aliquots of the samples were removed at 0, 24, 48, and 72 h and subjected to immunoblotting after contrifugation at 18,000×*g* for 20 min.

### Generation and isolation of R-LFAOs

Monomeric Aβ42 (50 µM) was incubated with 5% (2.5 µM) LFAO seed in 20 mM Tris pH 8.0 at 25°C for 72 h. After 72 h, the sample was subjected to SEC on a Superdex-75 HR 10/30 column after centrifugation at 18,000×*g* for 20 min to remove fibrils. SEC fractions 16 and 17 were collected and subjected to immunoblotting to confirm the presence of R-LFAOs.

### Dynamic light scattering (DLS)

DLS was performed on a Zetasizer Nano S DLS instrument (Malvern, Inc., Worcestershire, UK). Each sample measurements consisted of 6 runs of 10 s each with a pre equilibration time of 40 s. After the measurement, the number (%) was exported and plotted against size using the origin 7.0 software.

### Circular dichroism (CD)

CD spectra were obtained in the far UV region with a Jasco J-815 spectropolarimeter (Jasco Inc, Easton, MD). Samples were placed in a 0.1 cm path-length quartz cuvette (Hellma) and were monitored in continuous scan mode (260–190 nm). The acquisition parameters were 50 nm/min with 8 s response time, 1 nm bandwidth, and 0.1 nm data pitch, and data sets were averaged over two scans. Spectra of appropriate blanks were subtracted from data sets as indicated. The corrected, average spectra were smoothed using a ‘mean-movement’ algorithm with a convolution width of 25 using the Jasco spectra analysis program.

### Polyacrylamide gel electrophoreses (PAGE) and immunoblotting

Samples were dissolved in loading buffer (1x Laemmli buffer) containing 1% SDS, applied without heating to 4–12% NuPAGE gels (Invitrogen) containing bis-Tris, and resolved in MES running buffer with 0.1% SDS. Dye-linked MW markers (Blue Plus2 Prestained Standards, Invitrogen) were run in parallel for calibration. For immunoblotting, gels were electroblotted onto 0.45 µm Immobilon nitrocellulose membranes (BioTrace NT, Life Sciences Inc). Blots were boiled in a microwave oven in PBS for two min and were blocked overnight with 1X PBS containing 5% nonfat dry milk and probed (1–2 h) with 1∶1000–1∶2500 dilutions of monoclonal antibody Ab9 or Ab5, which detect amino acid residues 1–16 of Aβ. Blots were then incubated with anti-mouse horseradish peroxide (HRP) conjugate and developed with ECL reagent (Thermo Scientific). ADDLs and Aβ globulomers were visualized using silver stain after gel electrophoresis [29].

### Dot blot analysis

Samples (250 ng) were spotted onto a 0.45 µm Immobilon nitrocellulose membrane and allowed to dry at room temperature for 2 h. The blots were blocked for 4 h with 1X PBS containing 5% nonfat dry milk and probed overnight at room temperature with 1∶1000–1∶2500 dilutions of monoclonal antibodies Ab9 or Ab5 and also with polyclonal conformational specific antibody OC. Blots were then incubated with anti-mouse (for Ab9 and Ab5) or anti-rabbit (for OC) horseradish peroxide (HRP) conjugate and developed with ECL reagent (Thermo Scientific).

### Cellular NF-κB activation

Human neuroblastoma SH-SY5Y cells (American Type Culture Collection, Manassas, VA) were maintained (37°C, 5% CO_2_) in a 1∶1 mixture of Ham’s F12K medium and DMEM supplemented with 10% FBS, 100 units/mL penicillin, and 100 µg/mL streptomycin. Prior to experimentation, cells were seeded at a density of 5×10^5^ cells/mL onto 22×22 mm glass coverslips and allowed to adhere for 24 h. Aβ samples (monomer, LFAOs, and fibrils) prepared in 20 mM Tris-HCl (pH 8.0) were diluted 1∶8 into media containing 1% FBS media and added to cells at a final concentration of 0.5 µM Aβ. Cells treated with diluted buffer alone or 20 units/µL TNF-α served as negative and positive controls, respectively. Following 30 min treatment (37°C, 5% CO_2_), media was removed and cells were washed with PBS, fixed with 4% paraformaldehyde in PBS (10 min, 25°C), and permeabilized with 0.1% Triton X-100, 0.01 M glycine in PBS (10 min, 25°C). Fixed cells were blocked with 5% normal donkey serum, 1% BSA in PBS (10 min, 25°C) and incubated overnight (4°C) with mouse anti-NF-κB primary antibody (1∶600), which selectively binds to the activated form of NF-κB. Cells were rinsed, blocked with 5% normal donkey serum, 1% BSA in PBS (10 min, 25°C), and incubated (2 h, 25°C) with Alexa Fluor 555 goat anti-mouse, IgG conjugate secondary antibody (1∶1000). Stained coverslips were mounted on glass slides using Fluoroshield containing DAPI for nuclear staining. Labeled cells were imaged under a Zeiss LSM confocal microscope (Carl Zeiss, Thornwood, NY) using a PlanApo 63x/1.4 oil DIC objective. For each slide, three z-stack series were imaged using two-channel, 8-bit optical sections. Each z-stack consisted of 10 images taken at 0.42 µm intervals through the cell depth resulting in 0.0168 µm^3^ voxels per series. ImageJ software (NIH, Bethesda, MD) was utilized to export the LSM files into multi-frame TIFF images for analysis. Images shown are average intensity z-projections of one representative z-stack series. Custom MATLAB (MathWorks, Natick, MA) functions were developed to analyze the image data for each z-stack series. NF-κB activation (F) was defined as the sum of the pixel intensity values for all NF-κB channel pixels that were above a background threshold. To correct for varying cell population, NF-κB activation (F) was divided by the number of DAPI pixels above the background threshold. For each sample, the corrected NF-κB activation from each of the three z-stack series was averaged and normalized to the control. Results shown are the mean of three independent experiments, performed in triplicate.

### Statistical analysis

Dixon’s Q test was applied to the absorbance measurements for LFAO self-propagation to remove outliers with greater than 95% certainty. Quantitative increase was calculated by dividing the absorbance of the replicated LFAO’s SEC peaks by the averaged absorbance of at least three measurements of seed alone. Levene’s test (95% certainty) was used to examine homogeneity of variance among fold increase, and a two-way ANOVA followed by Tukey’s HSD (Honestly Significant Difference) was used to determine confidence intervals for each time/seed combination. A one-way ANOVA was also used to assess significance for NF-κB activation, and Dunnett’s test for multiple comparisons was used to identify groups with means significantly different from the negative control.

## Results

### LFAOs are self-propagating oligomers of synthetic Aβ42

**Figure 1:**
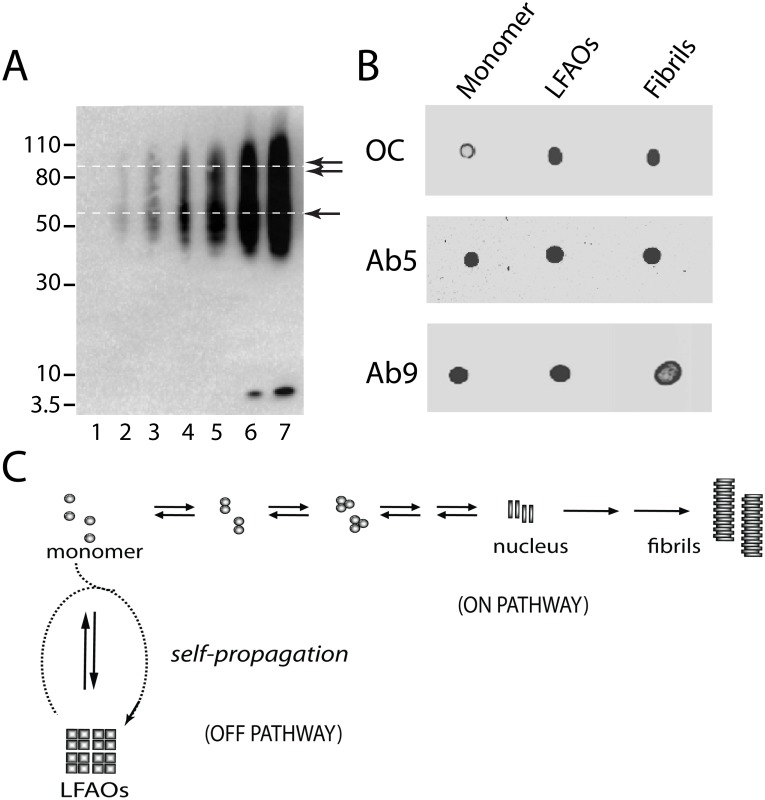
LFAO characterization. (A) Immunoblot showing different amounts of SEC-fractionated LFAOs. Lanes 1–7 correspond to 4, 19, 40, 80, 120, 250, & 500 ng of LFAOs, respectively. LFAOs show two distinct band distributions, a lower band centered around ∼56–70 kDa (single arrow) and an upper band between ∼80 and 110 kDa (double arrow) (B) Dot blot analysis of Aβ42 monomer, LFAOs, and fibrils probed using monoclonal antibodies OC, Ab5, and Ab9. The amount of sample used for dot blot was ∼250 ng and was kept constant for all the samples. (C) Schematic of LFAOs self-propagation reported earlier (27).

In our previous work, we demonstrated that size exclusion chromatography (SEC)-fractionated LFAOs include disperse oligomers of Aβ42 with a sedimentation coefficient ranging between 5 and 7 S and a corresponding molecular weight of 56–110 kDa (12–24 mer) [27]. As previously observed, immunoblots of SEC-isolated LFAOs indicated a disperse band between 56–110 kDa with two main band distributions, one centered at 56–70 and the other at 80–110 kDa (Fig. 1A) [27]. Although sedimentation velocity unambiguously established these molecular weight distributions within LFAOs [27], clearly, the visibility of the 80–110 kDa band in immunoblots depends on the amount of the sample loaded onto the gel (Fig. 1A). We have previously established, based on the nature of seeding and other biophysical criteria, that LFAOs are kinetically-trapped, fibril-like off-pathway products [26], [27]. To further probe the conformational characteristics of LFAOs, dot blot analysis was performed using the conformation-specific antibody OC, which is specific towards fibril-like oligomers [24] (Fig. 1B). LFAOs and fibrils both showed high reactivity towards OC antibody as compared to monomer, which displayed a weak reactivity as expected [30], confirming our earlier conclusion that LFAOs could be fibril-like conformers (Fig. 1B). The dot blot assay with the monoclonal antibodies Ab5 and Ab9, which have identical specificity towards the Aβ1-16 epitope [31], showed high reactivity towards all the samples (Fig. 1B). Both Ab5 and Ab9 have been used for immunodetection throughout this report. Although LFAOs did not show reactivity towards the oligomer-specific antibody A11, failure to generate A11-positive controls led us to interpret that the results were inconclusive (data not shown). Perhaps the most intriguing property of LFAOs, as demonstrated earlier [27], is their ability to replicate in the presence of monomeric Aβ42 (schematically shown in Fig. 1C). In other words, LFAO seeds are able to recruit Aβ42 monomers to generate quantitatively more LFAOs, which partly occurred at the expense of fibril formation implicating LFAOs to be a unique self-propagating strain of Aβ oligomers.

### LFAOs induce NF-κB activation in neuroblastoma cells

To evaluate the physiological response elicited by LFAOs, the ability of these oligomers to activate NF-κB in human neuroblastoma SH-SY5Y cells was assessed. This measure of physiological activity was selected because NF-κB immunoreactivity is elevated within Alzheimer’s brain and is co-localized with early plaque formation [32]. While the role of NF-κB in neuronal function is uncertain [33], NF-κB activation provides a clear indicator of the physiological effect of Aβ upon cellular function. Furthermore, NF-κB activation offers an advantage over measures of cell death, as NF-κB activation can be observed within 30 min of cellular treatment, thus diminishing the possibility that assembled Aβ structures are altered during incubation in cell culture media.

**Figure 2:**
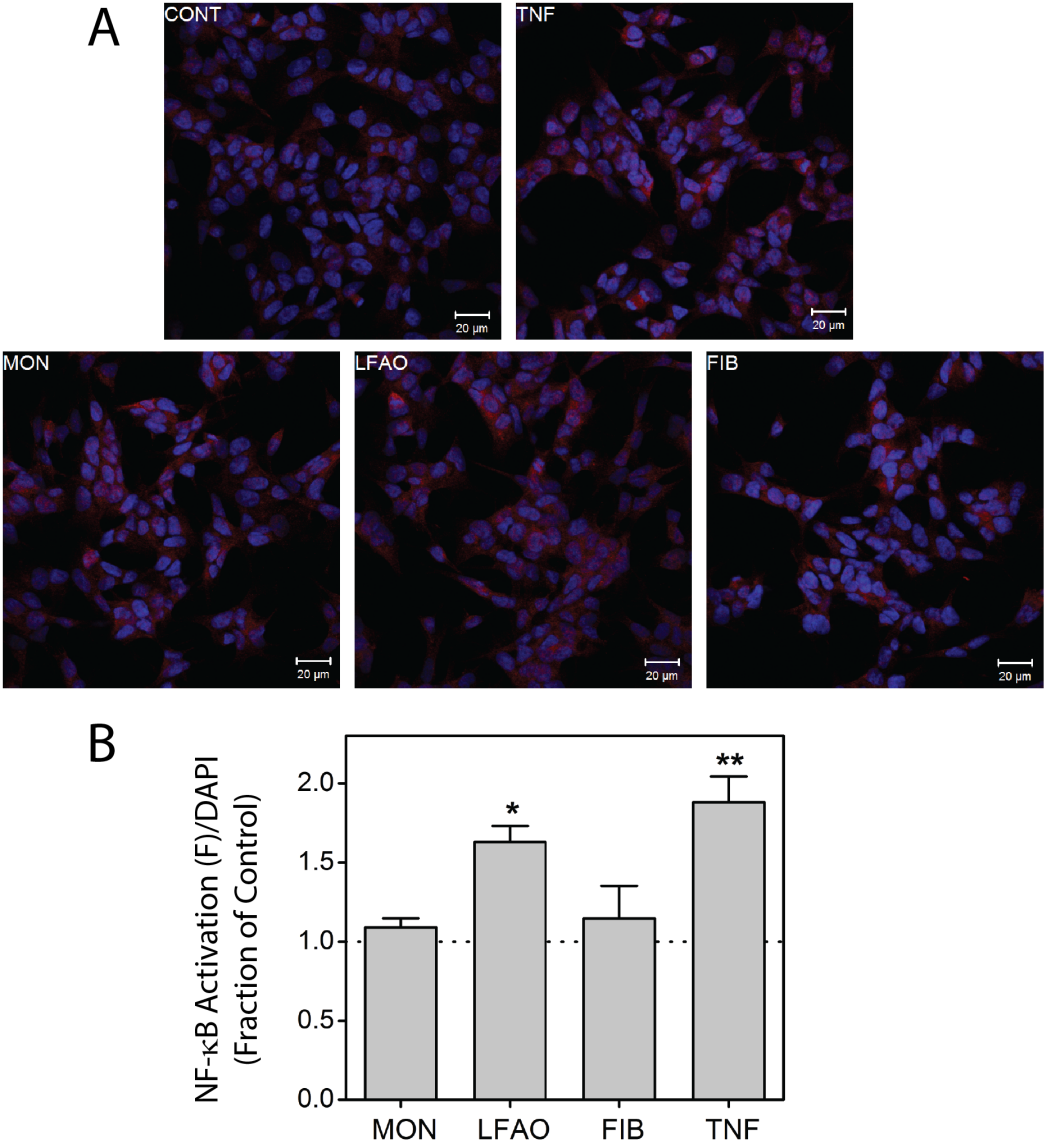
Effect of LFAOs on human SH-SY5Y neuroblastoma cells. (A) SH-SY5Y cells were incubated in the absence (control, CONT) or presence of 0.5 µM Aβ monomer (MON), LFAOs (LFAO), or fibrils (FIB) for 30 min. Parallel treatment with TNF-α (TNF) served as a positive control for NF-κB activation. Immunofluorescence staining was performed using nuclear DAPI (blue) in conjunction with an anti-NF-κB antibody (red) that selectively binds to the activated form of NF-κB. (B) NF-κB activation was quantified using MATLAB functions of original z-stack confocal images, normalized for nuclear volume, and reported relative to control. Error bars indicate SEM, n = 3. The symbols * and ** represent p<0.05 and p<0.01, respectively, relative to the negative control.

Activation of NF-κB was assessed using an antibody that recognizes an epitope overlapping the nuclear localization signal of the p65 NF-κB subunit to selectively bind the activated form of NF-κB. Untreated, quiescent SH-SY5Y cells display low levels of NF-κB activation (Fig. 2A). In contrast, when cells are exposed to 0.5 µM LFAOs for 30 min expression of activated NF-κB is increased (Fig. 2A) to a level significantly higher than that observed for the negative, untreated control (Fig. 2B). A similar increase in NF-κB activation is also observed for SH-SY5Y cells treated with TNF-α, known to stimulate NF-κB activation in neuronal cells [33], [34]. These results demonstrate that LFAOs can induce physiological activity in a neuronal cell culture model. In contrast, SH-SY5Y cells treated with either monomeric Aβ or mature Aβ fibrils fail to exhibit significant NF-κB activation (Fig. 2). Thus, NF-κB activation induced by LFAOs is specific for this aggregation state of Aβ.

### Seeding efficiency of LFAOs in replication

**Figure 3:**
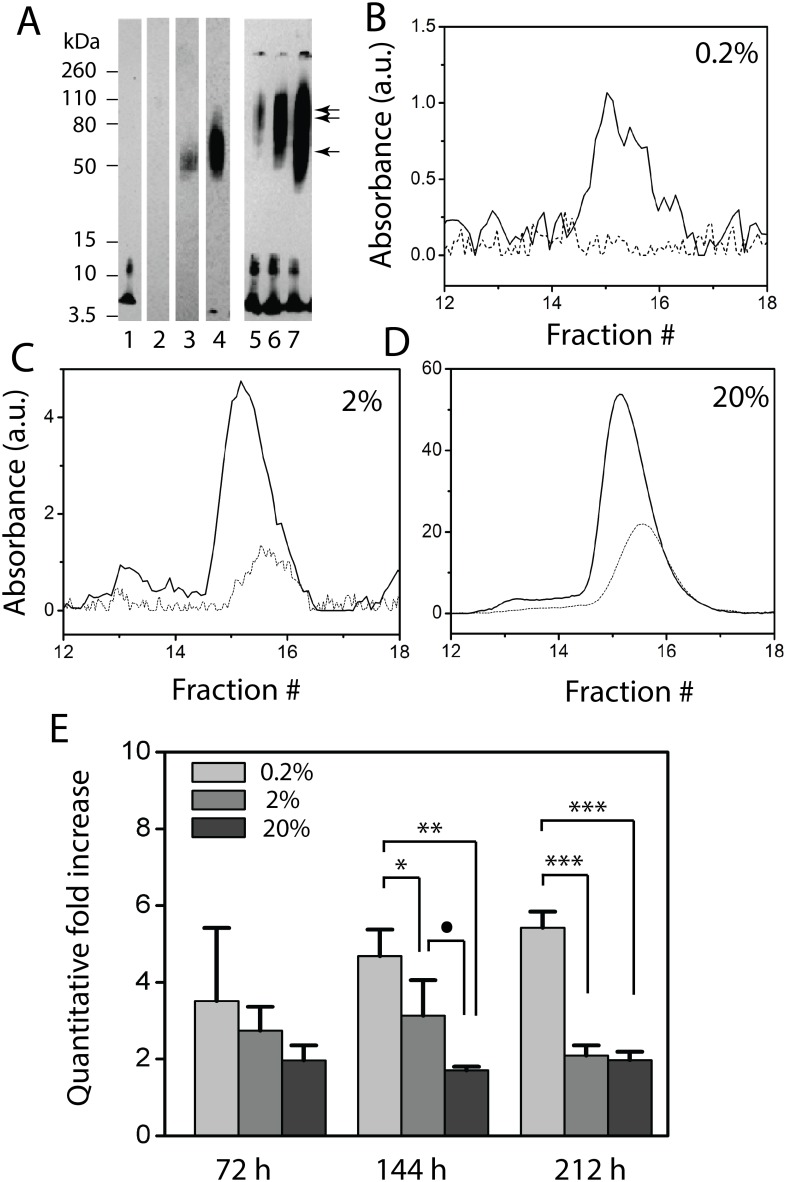
Determination of LFAOs seeding efficiency. (A) Immunoblot of 30 µM Aβ42 seeded with 0.2, 2, or 20% (molar ratio) LFAO seeds after 212 h. Lane 1 is the control Aβ42 incubated in the absence of seeds after 212 h. Lanes 2, 3, and 4 are 0.2, 2, or 20% (molar ratio) LFAO seeds alone, respectively. The amounts loaded in lanes 2, 3, and 4 are 4, 40, and 406 ng, respectively. Lanes 5, 6, and 7 are aliquots of 0.2, 2, and 20%-seeded reactions after 212 h, respectively. (B, C & D) Corresponding SEC chromatograms for the seeded reactions after 212 h. Solid lines and dashed lines correspond to seeded reactions and seed alone, respectively. (E) Quantitative increase of LFAO self-propagation for different seed amounts and at different time points. The data were obtained by normalizing the SEC chromatogram for the seeded reactions against the corresponding chromatograms of the seeds alone. The symbols •, *, **, and *** represent >90, >95, >99 and >99.9% confidence based on ANOVA statistical analysis.

In our previous report, the replication property of LFAOs was qualitatively established [27]. It has been reported that for self-propagation to occur the seed concentration of infectious prions must be higher than a minimum threshold concentration, below which propagation will not occur [35], [36]. A similar critical threshold concentration has been determined for fibril seeding for Aβ40 [37]. Therefore, in order to evaluate the efficiency of LFAO seeds to undergo replication, a sample containing freshly purified, seed-free 30 µM Aβ42 monomer in 20 mM Tris, pH 8.0, was incubated with 0.2, 2, and 20% (molar) LFAO seeds at 25°C, and the reaction was monitored up to 212 h. In addition to the presence of some monomers, 4–5 mers, and high molecular weight aggregates that failed to enter the gel as expected, the immunoblots of the reactions after 212 h also showed significant increase in the amounts of LFAOs (Fig. 3A, lanes 5–7) relative to the seed alone (Fig. 3A, lanes 2–4). In order to unambiguously determine the amount of LFAOs generated, the samples were fractionated through a Superdex-75 size exclusion column (GE Healthcare Inc). Aliquots of samples were removed at 72, 144 and 212 h and subjected to SEC to obtain quantitative estimates of the increase in the amount of LFAOs formed due to self-propagation. Typically, aliquots of the reactions at 212 h were centrifuged at 18,000×*g* to remove any residual fibrils, and their supernatants were fractionated, generating respective chromatograms for 0.2 (Fig. 3B), 2 (Fig. 3C), and 20% (Fig. 3D) seeded samples. Quantitative increases in LFAO amounts were calculated by normalizing the area under the curve (AUC) of the SEC peaks against that of the seed alone. Interestingly, increasing the percentage of seeds resulted in a quantitative decrease in LFAO formation (Fig. 3E). The most significant increase in the amount of LFAOs after 212 h was observed in the immunoblot for the 0.2% seeded sample (∼5.5-fold increase, light grey bar) followed by 2 and 20% seeds (∼2-fold increase, grey and dark grey bars), with the difference between the 0.2% seeded sample and either the 2 or 20% seeded sample being statistically significant (p<0.001). Identical analyses of the samples after 72 and 144 h of incubation also led to a similar trend in the increase of LFAOs, with the 0.2% seeded sample showing the maximum increase followed by the reactions seeded with 2 and 20% LFAOs (Fig. 3E). Reactions that were incubated for more than 212 h did not result in any further significant increase (data not shown). The reaction with 2% seeds showed a maximum increase (three-fold) at 144 h that decreased to less than two-fold after 212 h, while the reaction with 20% seeds showed only a two-fold increase throughout the incubation time (Fig. 3E). Attempts to characterize 0.02% seeded reactions resulted in signals in SEC and immunoblots that were insignificant and did not allow for statistically meaningful analysis (data not shown). We have previously established that LFAOs are fibril-like oligomers that are formed along an alternate pathway [27]. The fibril-like nature of LFAOs was also confirmed by their specificity for the conformation-specific OC antibody (Fig. 1B). Perhaps due to its fibril-like nature no significant difference between aggregation profiles by thioflavin-T assay was observed between seeded and non-seeded reactions (data not shown). We hypothesize that LFAO replication and fibril formation reactions compete with each other. For this same reason, it is likely that the replication appears more efficient at a lower seed percentage than the higher ones, as the presence of a higher amount of seed could favor seeding towards fibril formation than replication. A similar observation of increased propagation efficiency at higher dilutions was reported for PrP^Sc^ amplification [38].

### Effect of temperature on LFAO replication

**Figure 4:**
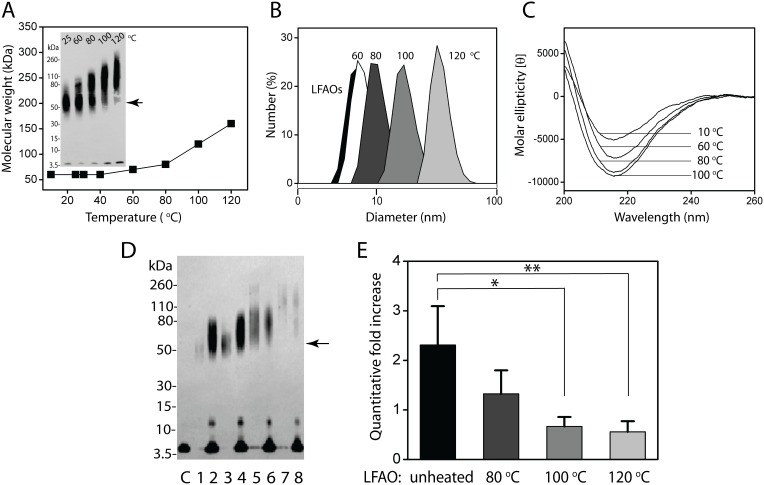
Effect of temperature on LFAOs and their self-propagation . (A) Data derived from the immunoblots show the increase in LFAO molecular weight with increase in temperature. *Inset*, immunoblot of LFAOs heated at the indicated temperatures; Lane 1 shows unheated LFAO sample (60 ng), while lanes 60, 80, 100, and 120°C show LFAOs heated-treated at these temperatures. Single arrow represents the center of unheated LFAOs molecular weight band. (B & C) DLS and CD analyses of the heat-treated LFAOs. D) Immunoblot comparing self-propagation of unheated LFAOs v/s those that were pre-treated with 72 h incubation at 80,100, and 120°C. Lanes 1, 3, 5, & 7 are the seeds alone (20 ng) for unheated, 80, 100, & 120°C-treated LFAOs (lanes 1, 3, 5 & 7, respectively). Lanes 2, 4, 6, and 8 represent 20 µM Aβ42 incubated with seeds of unheated, 80, 100, & 120°C-treated LFAOs, respectively after 72 h. E) Quantitative increase in LFAO amounts derived from seeded reactions and based on the normalization procedure used in Fig. 3. The symbols * and ** represent p<0.05 and p<0.01 based on ANOVA statistical analysis.

In an effort to optimize LFAO replication, the effect of temperature on LFAOs was investigated. Previous studies on PrP^Sc^ have shown that high temperatures promote the formation of larger aggregates that greatly reduce propagation efficiency [39]. To determine the behavior of LFAOs at high temperatures, buffered LFAOs (7 µM) were subjected to heating for 5 min at specific temperatures between 10 and 120°C. The samples were then allowed to equilibrate at 25°C for 15 min before immunoblotting and DLS analyses (Fig. 4A & B). No change in LFAO molecular weight was observed up to 50°C (data not shown), and a marginal increase in molecular weight (centered at 75 kDa) was observed at 60°C in the immunoblot as compared to the LFAOs at 25°C (centered at 56 kDa) (Fig. 4A; *inset*). It has to be borne in mind that two bands of LFAOs are apparent only when you increase the amount loaded (see Fig. 1A). A shift towards higher molecular weight along with noticeably intense banding was observed for samples heated at 80, 100, and 120°C (Fig. 4A). While the molecular weight of the gel band was centered at approximately 80 kDa for the sample treated at 80°C, the bands were centered at 110 and 160 kDa for samples heated at 100 and 120°C, respectively. A corresponding shift in molecular size was also observed for the same samples in DLS (Fig. 4B). The average hydrodynamic diameter of the monodisperse peaks displayed by the heat-treated LFAO samples at 60 (white), 80 (dark grey), 100 (grey), and 120°C (light grey) were centered at 8, 11, 18, and 37 nm, respectively. Except for the 60°C-treated sample, all heated sample diameters were considerably larger than the 7–8 nm diameter observed for the LFAO sample at 25°C (Fig. 4B; black peak). CD analysis also showed an increase in β-sheet content concomitant with the increase in size, characterized by a minimum at 216 nm in the far-UV region (Fig. 4C). This observation was consistent with the report by Gursky and colleagues [40], which showed that heating of Aβ40 at high temperatures caused an increase in β-sheet content, suggesting an increase in oligomerization and aggregation at elevated temperatures. A similar observation has also been reported for other β-sheet forming proteins such as barstar [41]. Together, the data indicate that increased temperatures promote the formation of larger aggregates of LFAOs, a behavior similar to PrP^sc^.

Next, the efficiency of self-propagative replication using LFAO seeds that were pre-heated at high temperatures, 80, 100, and 120°C, was investigated. Using LFAO seeds prepared as in Fig. 3, 20 µM freshly purified, aggregate-free Aβ42 was incubated with 2% (molar ratio) LFAOs (control and pre-heated). Fig. 4D shows the immunoblots of the reactions after 72 h. The band intensities for the reactions with unheated, 80, 100, and 120°C-heated LFAO seeds (20 ng; lanes 2, 4, 6, and 8, respectively, in Fig. 4D) suggest diminishing self-propagating efficiency with increasing temperature. Increases in band intensity with increase in temperature were consistent with those observed in Fig. 4A (*inset*). The quantitative increase, determined by comparing the SEC peaks of seeded sample with seed alone, indicated a ∼2.8 fold increase for the unheated LFAO sample (Fig. 4E, unheated), which is comparable to the data obtained previously (Fig. 3C). In contrast, the reactions with LFAO seeds pre-heated at 80, 100, and 120°C showed 1.4-, 0.7-, and 0.4-fold increases, respectively, (Fig. 4E), which were significantly lower than that for unheated LFAOs – indicating a decrease in propagation efficiency with increase in temperature. Statistical analysis (ANOVA) indicated p<0.05 (80°C; •) and p<0.01 (100 and 120°C; **) significance in the difference between the seeding efficiency of unheated and heated LFAOs after 72 h. A similar decrease in propagation with pre-heating was observed with PrP^Sc^ [39], which further suggests that LFAOs could be prion-like Aβ oligomers. It is also noteworthy that the diminished replication efficiency observed for LFAOs pre-heated to high temperatures can be attributed to the increase in molecular mass of LFAOs. Furthermore, even the larger aggregates seem to undergo replication (based on increase in the band intensity and the absence of fibrils). This observation reiterates that the process of self-propagative replication could compete with the fibril formation pathway, as we concluded in our previous work [27]. This also implicates that the replicative process could be limited to a few specific ‘conformational-strains’ of Aβ oligomers.

### Replication efficiencies of parent and R-LFAOs

**Figure 5:**
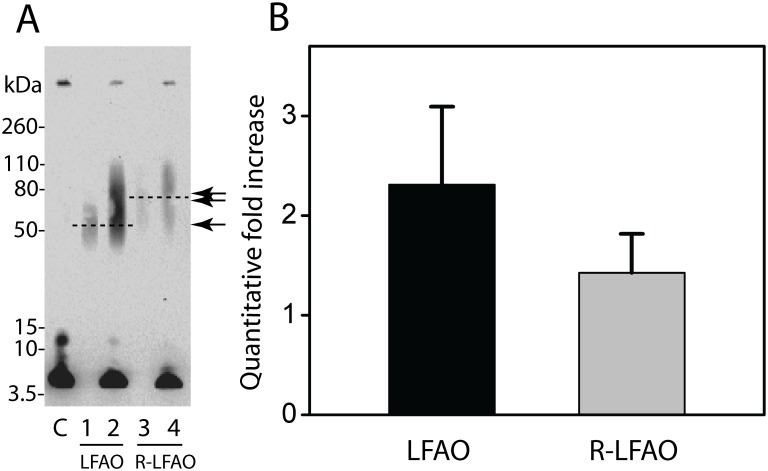
Comparison of propagation efficiency of parent LFAOs and R-LFAOs. (A) Immunoblot of LFAO and R-LFAO seeded sample after 96 h. ‘C’ represents the control sample in the absence of seed, lanes 1 and 2 show parent LFAO seed alone and seeded reaction after 96 h, respectively, while lanes 3 and 4 show R-LFAO seed alone and seeded reaction after 96 h, respectively. Single and double arrows indicate the parent LFAO seeds ∼56–70 kDa and formation of R-LFAO ∼80–110 kDa bands after propagation, respectively. (B) Comparison of quantitative fold increase after seeding with parent and R-LFAO.

In an effort to understand whether the lower molecular weight LFAOs (56–70 kDa) contribute to the efficiency of self-propagation preferentially over the larger ones (80–110 kDa) (Figs 1A & 3A, and [27]), the replication of R-LFAOs, which are predominantly higher molecular weight bands of LFAOs (80–110 kDa), was examined. R-LFAOs were generated as described (see Methods), with the exception that 5% (molar ratio) LFAO seed was used instead of 2%, as used for cyclic propagation experiments (Fig. 5). This practice was adopted to increase the quantitative yield of the SEC-purified R-LFAO seed to a level high enough to initiate the second propagation cycle and facilitate comparison of its propagation efficiency with that of parent LFAOs. Using either parent LFAOs or R-LFAOs as seeds, the LFAO self-propagation procedure was followed using 2% (molar ratio) seeds with 20 µM buffered Aβ42 monomer. A 96 h incubation time, rather than 72 h, was adopted for this experiment as better signals in immunoblots were obtained. The immunoblot of the reaction seeded with R-LFAO clearly shows diminished band intensity (lane 4) as compared to the reaction seeded with parent LFAO (lane 2), suggesting a decrease in the self-propagation efficiency (Fig. 5A). In addition, quantitative analysis by SEC showed a ∼2.3-fold (black bar) and a ∼1.4-fold (grey bar) increase for the LFAO and R-LFAO seeded reactions, respectively (Fig. 5B), which corresponds to a ∼33% decrease in propagation efficiency with R-LFAOs as seeds. The data suggest that the larger ∼80–110 kDa (18–24 mer) R-LFAOs are less efficient for replication by self-propagation as compared to parent LFAOs.

### Can other *in* *vitro* oligomers undergo replication by self-propagation?

**Figure 6:**
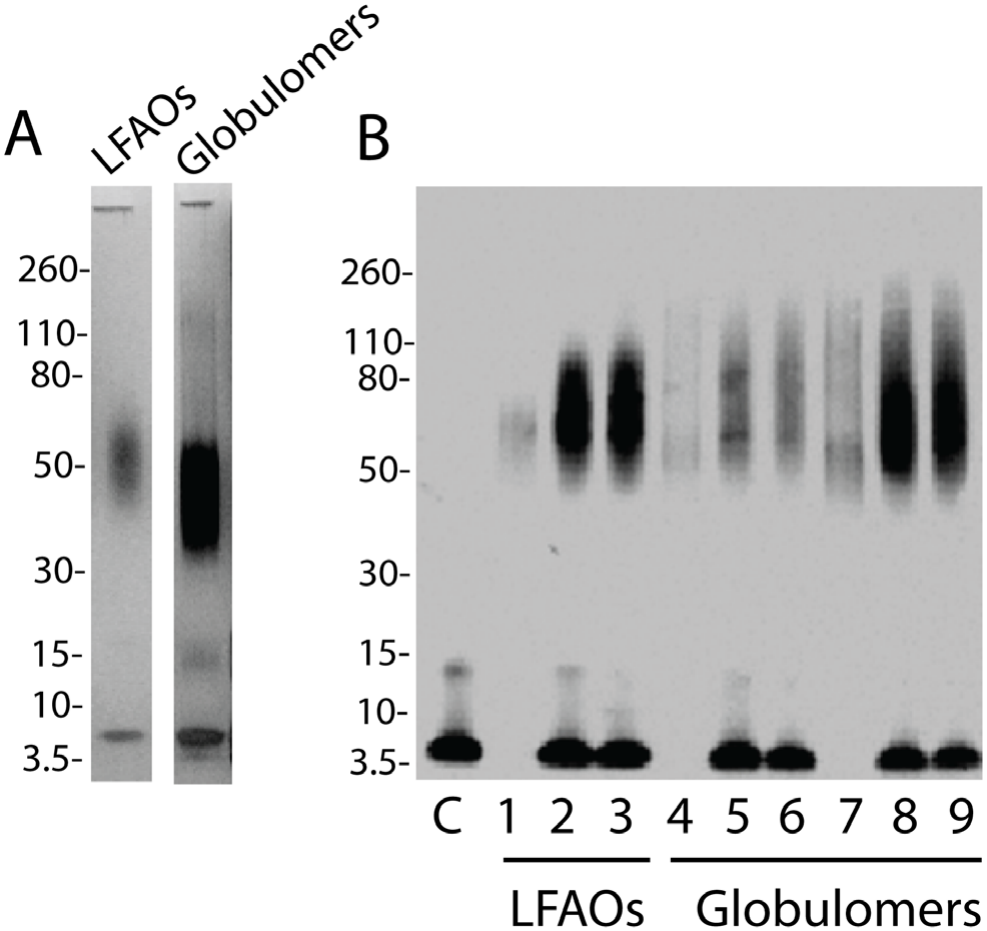
Comparison of seeding behavior of globulomers and LFAOs. (A) Silver stain of LFAOs and globulomers generated using previously established protocols. (B) Immunoblot showing the comparison of seeding efficiency of LFAOs and globulomers. Lane C represents Aβ42 control after 72 h. Lanes 1, 4, and 7 show 2% (molar ratio) LFAOs, 2 and 5% (molar ratio) globulomers seed alone, respectively. Lanes 2 and 3 show total and supernatant of LFAOs seeded sample after 72 h, while lanes 5, 6, 8, and 9 show total and supernatant of 2 and 5% globulomers seeded sample after 72 h, respectively.

In light of the replication behavior displayed by LFAOs, it is important to know whether this property is unique to LFAOs or whether other *in* *vitro* oligomers could also replicate. To answer this question, the neurotoxic Aβ globulomers (∼10–12 mers) [28] were subjected to undergo self-propagation similar to LFAOs. Globulomers were generated using previously published protocols and were confirmed by silver staining and immunblotting (Fig. 6A and 6B, respectively). Freshly purified Aβ42 monomer (20 µM in 20 mM Tris pH 8.0) was incubated at 25°C with 2 and 5% (molar ratio) globulomers for 72 h. A parallel reaction seeded with 2% (molar ratio) LFAO was used as a positive control. Globulomers showed a significant degree of self-propagation after 72 h, similar to LFAOs (Fig. 6B). Interestingly, the globulomer propagation efficiency increased when the percentage of seed used was increased from 2% (molar ratio), shown in lanes 5 and 6, to 5% (molar ratio), shown in lanes 8 and 9 (Fig. 6B). The immunoblot showed a large disperse band for globulomers ranging from ∼40–96 kDa after 72 h, which was comparable to LFAOs (Fig. 6B, lanes 2 and 3). However, based on band intensities, the 2% LFAO seeded self-propagation reaction (Fig. 6B, lanes 2 and 3) appeared more efficient than the 2% globulomer-seeded reaction (Fig. 6B, lanes 5 and 6). On the contrary, the band intensity of the 5% globulomer seeded reaction (Fig. 6B, lanes 8 and 9) was comparable to that of 2% LFAO seeded reaction (Fig. 6B, lanes 2 & 3). Interestingly, self-propagation of globulomers leads to the formation of larger disperse oligomeric band between 60 and 90 kDa instead of the 38–48 kDa discrete band observed in parent globulomers, an observation similar to the one for LFAO self-propagation. In sum, the data suggest that globulomers are able to undergo self-propagation upon monomer addition similar to LFAOs, indicating the possibility of structural or conformational similarity among the two. It is noteworthy that similar to LFAOs, globulomers are also identified as species formed along off-fibril formation pathway. Perhaps, this similarity is the likely reason for their comparable replication behavior. Our attempts to probe self-propagative replication of another well-known Aβ oligomer called, ADDLs remained inconclusive although largely no replication was observed (data not shown).

## Discussion

### Replication by self-propagation could be limited to a few conformational variants of Aβ oligomers

The findings presented in this report showcase the self-propagative replication of 12–24 mer oligomers of Aβ42 (LFAOs). The process of replication by self-propagation is different from the seeding of Aβ aggregates towards fibril formation. Although fibril formation also occurs via a template-assisted mechanism, it does not lead to the generation of homogenous, monodisperse species with a specific molecular mass. Instead, it leads to the formation of a continuum of species with varying molecular mass (fibrils are usually heterogeneous species with high molecular mass). Furthermore, this nucleation-dependent process remains dynamic until insoluble fibrils are formed. Replication by self-propagation, on the other hand, occurs when an oligomer of a specific molecular weight and conformation interacts with monomer to generate quantitatively more oligomers of similar molecular mass and conformation, which occurs, in part, at the expense of fibril formation (Fig. 1C).

**Figure 7:**
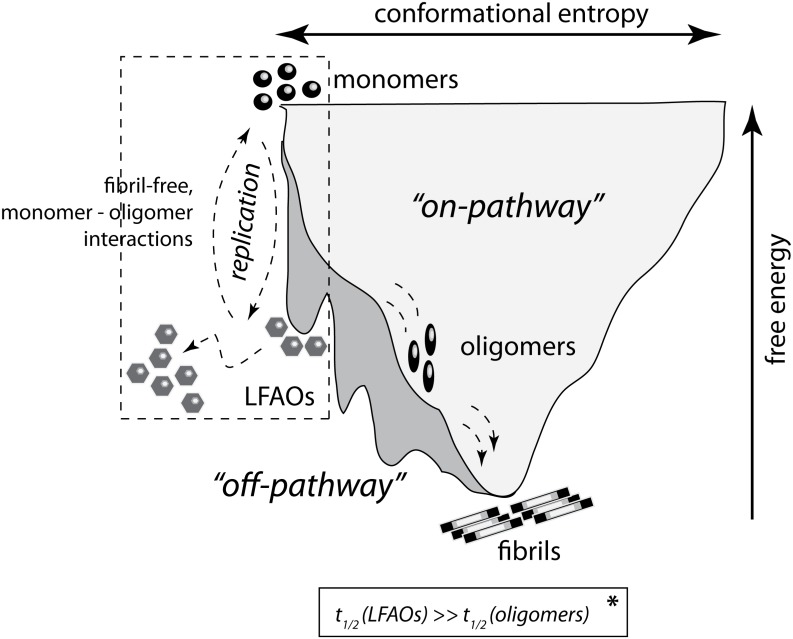
Schematic indicating the ‘aggregation funnel’ and self-propagative replication of LFAOs. LFAOs are kinetically-trapped, off-pathway intermediates due to which their half-lives could be considerably longer than the on-pathway oligomers (* indicates that the condition is hypothetical and not derived from the data presented in the manuscript). The second important trait of LFAOs is that they are conformationally distinct to undergo replication via fibril-free monomer – oligomer interactions as indicated.

Unlike prions, for which a conformational switch from the cellular PrP^C^ to scrapie PrP^SC^ is sufficient to trigger the self-propagation reaction, conformational change alone does not appear to be sufficient for replication by self-propagation among Aβ aggregates. This is because aggregation of Aβ generates a plethora of polydisperse and polymorphic aggregates due to the presence of multiple conformational variants. It seems likely that the property of self-propagation is a virtue of only certain conformational strains. This is clear from the fact that, although both LFAOs and protofibrils have significant β-sheet conformation, only the former is able to undergo replication; protofibrils are known to elongate upon monomer addition [42], [43]. Clearly, not all oligomers of Aβ possess the propensity to self-propagate, as our unpublished data on ADDLs seem to suggest (data not shown). We speculate that oligomers that are the intermediates along the on-pathway towards fibril formation are likely to seed fibrils in a nucleation-dependent manner and may not undergo self-propagation. One of the main distinctions between LFAOs and other pre-fibrillar intermediates such as protofibrils is that the LFAOs are formed as kinetically-trapped intermediates along the off-fibril formation pathway in the aggregation landscape [27] (Fig. 7). This may allow the oligomers to: *a*) possess longer half-lives than their on-pathway counterparts, which would transiently prevent fibril formation, and *b*) adopt a uniquely distinct conformation than those adopted by the on-pathway oligomers. Together, these properties make it conducive for the fairly homogenous and conformationally-distinct LFAOs to interact with monomers and self-propagate. It is likely that the unique conformational characteristics and the physiochemical nature of off-pathway LFAOs are key elements that dictate their replicative properties. Yet another piece of evidence in support of this hypothesis comes from our data on globulomers, which replicate similar to LFAOs (Fig. 6B). As pointed out earlier, globulomers also have a predominant β-sheet conformation and are formed as off-fibril formation pathway intermediates [28], [44], [45]. A detailed understanding of LFAO structure in the future will allow for a better determination of the specific structural elements responsible for self-propagative replication. The current work, along with our previous report [27], has unambiguously established that LFAOs can undergo replication by self-propagation, similar to the conversion of PrP^c^ to PrP^sc^.

### LFAOs are physiologically active

Previous observations on prion-type propagation of Aβ were reported based on experiments using transgenic mouse models, where isolated brain homogenates were inoculated to observe Aβ replication [5]–[7]. In a similar experiment, Stohr and colleagues inoculated transgenic mice with synthetic Aβ aggregates to observe identical prion-type behavior [11]. However, in these reports, a precise biophysical nature of the Aβ aggregates inoculated was not characterized. In this report, we have established that LFAOs are distinct oligomeric strain that can undergo self-propagative replication. In order to explore the physiological relevance of these propagating Aβ aggregates, we evaluated the activity of LFAOs toward SH-SY5Y human neuroblastoma cells. LFAOs were capable of activating NF-κB, while Aβ monomers and fibrils remained inert. Since NF-κB, a central regulator of inflammation, is highly activated in AD brains, this activation indicates that LFAOs are physiologically relevant oligomers of Aβ, and thus their propagation could contribute to disease pathogenesis.

## Conclusions

The findings reported here are significant in that they are a detailed demonstration of *in* *vitro* replication by self-propagation of Aβ oligomers. Investigation into the transmissible nature of self-propagating Aβ oligomers will be significant in furthering the existing understanding of the pathogenic mechanisms involved in AD and other amyloid diseases. Importantly, replication of soluble oligomers may hold profound implications since such a mechanism could manifest in transmissibility and dissemination of toxic species, as observed previously in transgenic animal models. Furthermore, the quantitative increase in Aβ oligomers by replication could also be useful in amplifying small amounts of endogenous seeds, using exogenous monomers, to quantities amenable for biophysical and structural characterization. Amplification may also facilitate the early detection of these self-propagating oligomeric, physiologically active species present in plasma and CSF, providing a potential biomarker for diagnostic purposes in AD.
